# LONELINESS, SOCIAL ENGAGEMENT, AND COGNITIVE STATUS AMONG OLDER BLACK AND WHITE ADULTS

**DOI:** 10.1093/geroni/igae098.3452

**Published:** 2024-12-31

**Authors:** Bettina Beech, Marino Bruce, Charles Udo, Ana Velazquez, Roland Thorpe

**Affiliations:** University of Houston, Houston, Texas, United States; University of Houston, Houston, Texas, United States; University of Houston, Houston, Texas, United States; University of Houston, Houston, Texas, United States; Johns Hopkins University, Baltimore, Maryland, United States

## Abstract

Social connection has been consistently shown to influence health status and recent research findings suggest that an absence of relationships with significant others can increase risks for cognitive impairment. Few studies have examined how the presence or absence of social connections have implications for cognitive impairment and even fewer have considered how these associations can vary by race and gender. The purpose of this study was to examine race and gender differences in the associations between loneliness, social engagement, and cognitive impairment among Black and White adults in the Health and Retirement Study (HRS). Data were drawn from participants who completed the Core and Leave Behind Questionnaire items in the 2016 HRS. Any cognitive impairment, a dichotomous variable derived from the Telephone Interview for Cognitive Status, was the outcome of interest. Loneliness and social engagement were the primary independent variables. Black men (35.5%) and Black women (27.0%) were disproportionately represented among respondents with any cognitive impairment relative to White men (14.6%) and White women (16.2%). Results from race- and gender-stratified Modified Poisson Regression models indicated that loneliness was associated with a higher prevalence of any cognitive impairment (PR=1.29, CI:1.07-1.57) among White women. Social engagement was inversely related with any cognitive impairment among White men (PR=0.97, CI:0.95-0.99). Neither of the main independent variables were significant for Black men or women in the study. Studies with robust and inclusive social connection measures are needed to further examine the association between loneliness, social engagement, and cognitive functioning among diverse populations.

